# Supplementary material for: Associations between Delayed Introduction of Complementary Foods and Childhood Health Consequences in Exclusively Breastfed Children
Source: Nutrients. 2023 Jul 31;15(15):3410. doi: 10.3390/nu15153410 (PMC10421357; doi:10.3390/nu15153410)
Supplement: Supplementary file 1 [file nutrients-15-03410-s001.zip › nutrients-2493340-supplementary.pdf]

Supplementary Table S1. Operating characteristics of diagnosis codes used to define primary outcome diseases of interest.

Outcome[a]	Definition	ICD-10 codes used in the study
Pneumonia[1]	Inpatient visit with a pneumonia code	J12; J 15; J18
Asthma [2]	Outpatient hospital visit with an asthma code of J45 (asthma), J46 (status asthmaticus) and ≥1 claim as an inpatient.	J45; J46
URTI [3]	Inpatient visit with an acute upper respiratory infections code	J00-J03; J06
Acute gastroenteritis [4]	Inpatient visit with an intestinal infectious diseases code	A00-A09
Acute otitis media	Any hospital visit with an acute otitis media code with relevant procedure codes	H65.X, H66.X, H67.X
Tooth decay	Any hospital visit with decayed, missing, or filled teeth	K02.X
ADHD[b] [5]	Individuals who received a diagnosis of ADHD with at least two records of psychiatric outpatient visits within one year or one admission diagnosis.	F90
Autism spectrum disorder[c] [5]	Two or more in-/outpatient visit with autism spectrum disorder codes for at least 6 months interval	F84.0, F84.1, F84.9
Epilepsy [5]	Inpatient visit with an epilepsy code or ≥ 2 hospital visits with an epilepsy code and two prescriptions of the same AED within six months.	G40; G41
Malignancy	Any in-/outpatient visit with a neoplasm code	C00-C97; D00-D48

Abbreviation: ADHD, Attention-Deficit/Hyperactivity Disorder; ICD-10, International Classification of Disease 10th Version; URTI, Upper Respiratory Tract Infection. [a] Subsequent clinical medical outcomes recorded after 24 months were investigated, unless otherwise stated. [b] Diagnosis recorded after 48 months of age in the national health insurance database was investigated. [c] Diagnosis recorded after 18 months of age in the national health insurance database was investigated.

Supplementary Table S2. Variables in the propensity score matching of the main cohort [a].

Characteristic	Database	Variables
Demographics	NHIS	Age, sex, income quintile, residence at birth
	NHSPIC[b]	Birth weight, types of feeding (breastmilk, formula, mixed, or special milk) during 4-6 months of life, whether introduced to solid food before 4 months of life, weight at 4-6 month, head circumference at 4-6 month, weight gain during the first 4-6months
Use of hospital resources	NHIS	Visit to a pediatrician, visit to the Emergency Room (ER)
Physical examinations	NHSPIC[b]	General condition, head and fontanelle, face, eyes, nose, ears, oral cavity/neck, lungs, heart, abdomen, genitals, extremities, spine, nerves, and skin
Supervision regarding child safety and sleeping habits	NHSPIC[b]	Parent answers to relevant questions
Comorbidities		
Health Care Use	NHIS	any hospitalization, ICU admission, death
Disease exposure before index date	NHIS (ICD-10 code)	
	Z38.0	Singleton, born in hospital
	J00	Acute nasopharyngitis
	J20.9	Acute bronchitis
	J06.9	Acute upper respiratory infection, unspecified
	J21.9	Acute bronchiolitis, unspecified
	P59.9	Neonatal jaundice, unspecified
	J02.9	Acute pharyngitis, unspecified
	L20.9	Atopic dermatitis
	A09.0	Other and unspecified gastroenteritis and colitis of infectious origin
	A09.9	Gastroenteritis and colitis of unspecified origin
	R50.9	Fever, unspecified
	L22	Diaper dermatitis
	L23.9	Allergic contact dermatitis
	J03.9	Acute tonsillitis
	J30.4	Allergic rhinitis
	L30.9	Dermatitis
	H10.3	acute conjunctivitis
	K52.9	Noninfective gastroenteritis and colitis, unspecified
	H10.9	Conjunctivitis, unspecified
	P38	Omphalitis of newborn with or without mild haemorrhage
	J18.9	Pneumonia, unspecified
	J45.9	Other and unspecified asthma

H66.0	Acute suppurative otitis media
K59.0	Constipation
J0.60	Acute laryngopharyngitis
A08.4	Viral intestinal infection, unspecified
J03.9	Acute tonsillitis, unspecified
J06.8	Other acute upper respiratory infections of multiple sites
J01.9	Acute sinusitis, unspecified
H66.0	Acute suppurative otitis media without spontaneous rupture of ear drum, unilateral or unspecified
N39.0	Urinary tract infection, site not specified
L20.8	Other atopic dermatitis
K59.9	Functional intestinal disorder, unspecified
H66.9	Otitis media, unspecified, unilateral or unspecified
J01.9	Acute sinusitis, unspecified, not specified as recurrent
L24.9	Irritant contact dermatitis, unspecified cause
P39.1	Neonatal conjunctivitis and dacryocystitis
B37.0	Candidal stomatitis
L21.1	Seborrhoeic infantile dermatitis
J30.3	Other allergic rhinitis
K21.9	Gastro-oesophageal reflux disease without oesophagitis
J22.0	Unspecified acute lower respiratory infection
L219	Seborrhoeic dermatitis, unspecified
K30	Functional dyspepsia
L50.9	Urticaria, unspecified
R11	Nausea and vomiting
J04.0	Acute laryngitis
J18.0	Bronchopneumonia, unspecified
K59.1	Functional diarrhea

Abbreviations: National Health Insurance Service, NHIS; National Health Screening Program for Infants and Children, NHSPIC. [a] Assessed from the date of birth to the index date, unless otherwise specified. [b] Obtained by the NHSPIC results performed at the first 4-6 months.

Supplement Table S3. Anthropometric measures and hospital visits compared by groups (4-<7 months *vs* ≥7 months) [a].

Measures, mean (SD)[b]	Observed Data (N=206,248)			Weighted Data (N =199,952) [c]		
	Non-exposed (n=165,925)	Exposed (n=40,323)	SMD,% [d]	Non-exposed (n=160,897)	Exposed (n=39,055)	SMD, %[d]
Birth weight, kg	3.23 (0.32)	3.23 (0.32)	2	3.23 (0.32)	3.23 (0.32)	0
Weight at 4-6 month [d]	8.00 (0.95)	8.09 (1.00)	10	8.02 (0.96)	8.02 (0.96)	0
Head circumference at 4-6 month [d]	42.57 (1.44)	42.63 (1.44)	4	42.58 (1.43)	42.58 (1.44)	0
Weight gain [e]	4.77 (0.90)	4.86 (0.92)	10	4.79 (0.9)	4.79 (0.9)	0
Visit to a pediatrician	7.53 (6.07)	7.64 (6.23)	2	7.55 (6.09)	7.55 (6.14)	0
Visit to the Emergency Room[f]	10,734 (6.47)	2,626 (6.51)	0	10,583 (6.58)	2,569 (6.58)	0

Abbreviations: SD, Standard deviation; SMD, Standardized mean difference. [a] Assessed from the date of birth to the index date, unless otherwise specified. [b] Values are reported as mean (SD) unless otherwise indicated. [c] Inverse probability of treatment weighting on the propensity score was used to balance comparison groups on baseline health. Covariates for weighting were selected *a priori* (Table 1, Supplementary Table 3-6). Individuals of the reference group were weighted with stabilized weights, which allowed producing a sample with the same distribution of the covariates as the exposed group. [d] Performed at the first 4-6 months. [e] Calculated by subtracting the birth weight from the weight measured at the screening program round 1(Weight measured between 4-6 months of age–birth weight). [f] Values are reported as number of participants (%).

Supplementary Table S4. Clinical characteristics of children compared by groups with different durations of exclusive breast milk feeding (4-<7 months *vs* ≥7 months) [a].

Clinical characteristics, N (%) [c]	Observed Data (N=206,248)			Weighted Data (N =398,037) [b]		
	Non-exposed (n=165,925)	Exposed (n=40,323)	SMD,% [d]	Non-exposed (n=160,897)	Exposed (n=39,055)	SMD, % [d]
Singleton, born in hospital	139,515 (84.08)	33,465 (82.99)	3	168,479 (84.68)	168,590 (84.69)	0
Acute nasopharyngitis	67,827 (32.12)	16,300 (40.42)	1	82,798 (41.61)	82,931 (41.66)	0
Acute bronchitis	53,299 (29.66)	13,581 (33.68)	4	65,876 (33.11)	65,858 (33.08)	0
Acute upper respiratory infection, unspecified	49,214 (18.67)	11,854 (29.40)	4	60,146 (30.23)	60,184 (30.23)	0
Acute bronchiolitis, unspecified	30,975 (18.67)	8,007 (19.86)	3	38,389 (19.29)	38,442 (19.31)	0
Neonatal jaundice, unspecified	38,756 (23.36)	9,546 (23.67)	1	47,511 (23.88)	47,609 (23.92)	0
Acute pharyngitis, unspecified	29,393 (17.71)	7,045 (17.47)	1	35,857 (18.02)	35,821 (17.99)	0
Atopic dermatitis	26,514 (15.98)	6,496 (16.11)	1	32,096 (16.13)	32,094 (16.12)	0
Other and unspecified gastroenteritis and colitis of infectious origin	15,100 (9.10)	3,497 (8.67)	2	18,350 (9.22)	18,411 (9.25)	0
Gastroenteritis and colitis of unspecified origin	13,899 (8.38)	3,191 (7.91)	2	16,823 (8.46)	16,837 (8.46)	0
Fever, unspecified	11,180 (6.74)	2,708 (6.72)	0	13,666 (6.87)	13,718 (6.89)	0
Diaper dermatitis	15,285 (9.21)	3,557 (8.82)	1	18,471 (9.28)	18,543 (9.31)	0
Allergic contact dermatitis	13,230 (7.97)	3,072 (7.62)	1	15,893 (7.99)	15,891 (7.98)	0
Acute tonsillitis	7,318 (4.41)	1,859 (4.61)	1	8,956 (4.50)	8,936 (4.49)	0
Allergic rhinitis	8,684 (5.23)	2,172 (5.39)	1	10,684 (5.37)	10,664 (5.36)	0
Dermatitis	11,214 (6.76)	2,643 (6.55)	1	13,539 (6.80)	13,592 (6.83)	0
acute conjunctivitis	9,371 (5.65)	2,260 (5.60)	0	11,387 (5.72)	11,404 (5.73)	0
Noninfective	8,163 (4.92)	1,852 (4.59)	2	9,827 (4.94)	9,824 (4.93)	0

gastroenteritis and colitis, unspecified						
Conjunctivitis, unspecified	8,917 (5.37)	2,232 (5.54)	1	10,905 (5.48)	10,953 (5.50)	0
Omphalitis of newborn with or without mild haemorrhage	9,396 (5.66)	2,226 (5.52)	1	11,454 (5.76)	11,468 (5.76)	0
Pneumonia, unspecified	3,328 (2.01)	922 (2.29)	2	4,183 (2.10)	4,179 (2.10)	0
Other and unspecified asthma	5,500 (3.31)	1,438 (3.57)	1	6,814 (3.42)	6,806 (3.42)	0
Acute suppurative otitis media	5,888 (3.55)	1,471 (3.65)	1	7,160 (3.60)	7,186 (3.61)	0
Constipation	8,801 (5.30)	2,011 (4.99)	1	10,628 (5.34)	10,597 (5.32)	0
Acute laryngopharyngitis	6,997 (4.22)	1,730 (4.29)	0	8,581 (4.31)	8,616 (4.33)	0
Viral intestinal infection, unspecified	6,416 (3.87)	1,413 (3.50)	2	7,715 (3.88)	7,745 (3.89)	0
Acute tonsillitis, unspecified	6,764 (4.08)	1,738 (4.31)	1	8,373 (4.21)	8,407 (4.22)	0
Other acute upper respiratory infections of multiple sites	5,970 (3.60)	1,456 (3.61)	0	7,311 (3.67)	7,342 (3.69)	0
Acute sinusitis, unspecified	4,841 (2.92)	1,177 (2.92)	0	5,892 (2.96)	5,885 (2.96)	0
Acute suppurative otitis media without spontaneous rupture of ear drum, unilateral or unspecified	5,449 (3.28)	1,403 (3.48)	1	6,762 (3.40)	6,737 (3.38)	0
Urinary tract infection, site not specified	2,617 (1.58)	644 (1.60)	0	3,206 (1.61)	3,206 (1.61)	0
Other atopic dermatitis	4,720 (2.84)	1,072 (2.66)	1	5,562 (2.80)	5,499 (2.76)	0
Functional intestinal disorder, unspecified	4,859 (2.93)	1,123 (2.79)	1	5,883 (2.96)	5,872 (2.95)	0
Otitis media, unilateral or unspecified	4,859 (2.93)	1,305 (3.24)	2	6,094 (3.06)	6,119 (3.07)	0
Acute sinusitis, not specified as recurrent	4,773 (2.88)	1,296 (3.21)	2	6,001 (3.02)	6,000 (3.01)	0

Irritant contact dermatitis, unspecified cause	5,332 (3.21)	1,289 (3.20)	0	6,461 (3.25)	6,477 (3.25)	0
Neonatal conjunctivitis and dacryocystitis	4,599 (2.77)	1,078 (2.67)	1	5,590 (2.81)	5,655 (2.84)	0
Candidal stomatitis	3,481 (2.10)	909 (2.25)	1	4,323 (2.17)	4,297 (2.16)	0
Seborrhoeic infantile dermatitis	4,723 (2.85)	1,090 (2.70)	1	5,682 (2.86)	5,701 (2.86)	0
Other allergic rhinitis	3,588 (2.16)	985 (2.44)	2	4,507 (2.27)	4,577 (2.30)	0
Gastro-oesophageal reflux disease without oesophagitis	3,490 (2.10)	800 (1.98)	1	4,219 (2.12)	4,234 (2.13)	1
Unspecified acute lower respiratory infection	3,041 (1.83)	771 (1.91)	1	3,751 (1.89)	3,732 (1.87)	0
Seborrhoeic dermatitis	4,216 (2.54)	1,061 (2.63)	1	5,134 (2.58)	5,173 (2.60)	0
Functional dyspepsia	3,612(2.18)	836 (2.07)	1	4,378 (2.20)	4,355 (2.19)	0
Urticaria, unspecified	3,651 (2.20)	897 (2.22)	0	4,455 (2.24)	4,441 (2.23)	0
Nausea and vomiting	2,707 (1.63)	599 (1.49)	1	3,255 (1.64)	3,293 (1.65)	0
Acute laryngitis	2,812 (1.69)	742 (1.84)	1	3,491 (1.75)	3,527 (1.77)	0
Bronchopneumonia	1,393 (0.84)	386 (0.96)	1	1,744 (0.88)	1,742 (0.88)	0
Functional diarrhea	2,863 (1.73)	690 (1.71)	0	3,502 (1.76)	3,498 (1.76)	0

[a] Assessed from the date of birth to the index date, unless otherwise specified. [b] Inverse probability of treatment weighting on the propensity score was used to balance comparison groups on baseline health. Covariates for weighting were selected *a priori* (Table 1, Supplementary Table 3-6). Individuals of the reference group were weighted with stabilized weights, which allowed producing a sample with the same distribution of the covariates as the exposed group. [c] Values are reported as No. (%) unless otherwise indicated. [d] The difference between the groups divided by the pooled standard deviation; a value greater than 10% is interpreted as a meaningful difference.

Supplement Table S5. Hospitalization related Diagnosis in children with ≥6 hospitalization events.

	Weighted Data (N =8,809) [a]	
	**Duration of Exclusive Breastfeeding, N (%)** [b]	
	**4-<7months (n=4,079)**	**≥ 7 month (n=4,730)**
Pneumonia	2,046 (50.16)	2441 (51.61)
Acute bronchitis, unspecified	1,320 (32.36)	1610 (34.04)
Acute tonsillitis, unspecified	1,280 (31.38)	1466 (30.99)
Influenza with other respiratory manifestations, seasonal influenza virus identified	987 (24.20)	1256 (26.55)
Other and unspecified gastroenteritis and colitis of infectious origin	904 (22.16)	1049 (22.18)
Bronchopneumonia,	801 (19.64)	990 (20.93)
Gastroenteritis and colitis of unspecified origin	791 (19.39)	930 (19.66)
Pneumonia due to Mycoplasma pneumoniae	737 (18.07)	902 (19.07)
Influenza with other respiratory manifestations, virus not identified	589 (14.44	682 (14.42)
Acute pharyngitis, unspecified	534 (13.09)	659 (13.93)
Acute bronchiolitis, unspecified	357 (8.75)	416 (8.79)
Enteroviral vesicular stomatitis with exanthema	280 (6.86)	384 (8.12)
Asthma, unspecified	280 (6.86)	377 (7.97)
Enteroviral vesicular pharyngitis	283 (6.94)	328 (6.93)
Fever, unspecified	279 (6.84)	327 (6.91)
Viral intestinal infection, unspecified	232 (5.69)	283 (5.98)
Acute obstructive laryngitis [croup]	210 (5.15)	257 (5.43)
Influenza due to identified zoonotic or pandemic influenza virus	194 (4.76)	256 (5.41)
Febrile convulsions	193 (4.73)	244 (5.16)
Rotaviral enteritis	190 (4.66)	217 (4.59)

[a] Participants the weighted cohort with ≥6 all-cause hospitalization events after 24 months of age. [b] Results are reported as n (%) unless otherwise indicated.

REFERENCE

1. Shin EJ, Kim Y, Jeong JY, Jung YM, Lee MH, Chung EH. The changes of prevalence and etiology of pediatric pneumonia from National Emergency Department Information System in Korea, between 2007 and 2014. Korean J Pediatr. 2018;61(9):291-300.
2. Lee SW, Yon DK, James CC, Lee S, Koh HY, Sheen YH, et al. Short-term effects of multiple outdoor environmental factors on risk of asthma exacerbations: Age-stratified time-series analysis. J Allergy Clin Immunol. 2019;144(6):1542-50 e1.
3. Shin SM, Shin JY, Kim MH, Lee SH, Choi S, Park BJ. Prevalence of antibiotic use for pediatric acute upper respiratory tract infections in Korea. J Korean Med Sci. 2015;30(5):617-24.
4. Ryoo E. Causes of acute gastroenteritis in Korean children between 2004 and 2019. Clin Exp Pediatr. 2021;64(6):260-8.
5. Ha EK, Lee SW, Kim JH, Shim S, Kim YH, Song JY, et al. Neurodevelopmental Outcomes in Infants Fed with Soy Formula: A Retrospective, National Population-Based Observational Cohort Study. J Nutr. 2021;151(10):3045-52.
